# Twelve Tips to implement Curriculum Changes in times of Economic Austerity

**DOI:** 10.15694/mep.2017.000047

**Published:** 2017-03-09

**Authors:** Kay Cartwright, Paladugu Madhavi, Keiarash Kazemi-Jovestani, Susan Maxwell, Maggie Wilson, Alexander Woywodt

**Affiliations:** 1 Lancashire Teaching Hospitals NHS Foundation Trust

**Keywords:** curriculum

## Abstract

This article was migrated. The article was marked as recommended.

Curriculum change is a recurring challenge facing most educational teams. Economic austerity has an impact on these processes in that clinical workloads increase and additional funds to drive curriculum change are lacking. We faced significant challenges having to implement substantial changes to the Year 3 and 4 undergraduate curricula in a large teaching hospital in the United Kingdom. The changes are now implemented successfully and we have taken the opportunity to identify factors that allowed us to drive change and achieve our aims. Much has been written about curriculum change as an academic challenge but comparatively little is known about how to drive such change on the ground and strategies to drive curriculum change during times of ongoing financial austerity are lacking. Here, we reflect on our experience and provide tips for educational teams on how to turn change into an opportunity, despite economic austerity and ever-increasing clinical workload.

## Introduction

In a world of constant change, undergraduate medical education is no exception. In the United Kingdom (UK) substantial changes to medical curricula occurred after the General Medical Council (GMC) published the first edition of Tomorrows Doctors in 1993 ( GMC, 2009). The intention of the original document was to better prepare undergraduates for their roles as junior doctors and recent studies suggest that this aim has been achieved to some degree ( Lewington, 2012). In a parallel development, the introduction of the National Students Survey (NSS) in 2005 required Universities throughout the UK to provide their Undergraduates with an ‘enhanced’ experience. This development has undoubtedly driven development of curricula throughout the UK. Our institution, a large teaching hospital in the North West of England, has been a base for Manchester Medical School (MMS) Undergraduates since 2003 and we now provide clinical training in Years 3, 4 and 5 of the MbChB course to 280 students in total. As an organisation, we are required to implement curriculum change on a very regular basis, first through the introduction a few years ago of a new curriculum for Year 4 of the course and more recently by the complete overhaul of the curriculum in Year 3 in 2016. We recognised early on that providing exciting, innovative timetables and motivating clinical staff who are already working within a constantly pressured clinical environment would be challenging. The changes are now well implemented but we felt that it would be interesting to reflect on our experience, not from the academic, scientific and scholarly point of view but looking at how change was driven and achieved in a health system under pressure. Another aspect of the challenge is that, at least in our health system, most educational leaders, such as Hospital Deans, Associate Deans, Academic Leads, lack formal training in management or organisational development. We speculate that an increasing number of educational teams are now faced with the dilemma of having to implement substantial curriculum change in an increasingly pressurised health service, without additional funding, and despite a lack of managerial training of the educational leadership team. Here, we reflect on the experience and provide a toolkit for educational teams that should enable them to see change as an opportunity rather than a threat, despite ongoing financial austerity.

## 12 Tips

### Tip 1: Scope the need for change, communicate the vision and identify opportunities

Teams vary in the way they react to substantial change on the horizon and some team members (or entire teams) may perceive change as a challenge or threat. This is especially true in times of austerity as team members will, either openly or not, wonder whether the change may make their own role redundant, increase their workload, or at the very least change the way of work they are accustomed to. The latter is particularly true if change involves changing the team culture ( Stewart, 1999) or introducing new ways of working. Early meetings should exclude any detail of the new curriculum but scope the extent of change and identify key strengths, opportunities and risks. Based on our experience with curriculum change, a year to eighteen months in advance is a good time to start these discussions. The early planning meetings eventually need to lead to what John Adair (2011) would identify as *‘real conversation’ with f*ace to face meetings were with all stakeholders and a purposeful discussion to move forward quickly.

It is also vital for the leadership team to have a clear idea of the scope of change and to communicate the vision to all team members and stake holders i.e. managers and clinical departments. Good communication and a clear idea of the vision will also provide reassurance to team members who feel threatened by the change and will ideally allow the leadership team to visualise the transformational change required and to “capture hearts and minds” ( Harshak, Aguirre, & Brown, 2010).

This early scoping period is also a great time to think out of the box and identify new opportunities. This may include areas or departments that have not taken students previously, clinicians who are not yet participating in undergraduate education or even opportunities in the community such as general practice or nursing homes. In our experience it is very valuable at this stage that all team members feel empowered and safe to float even unconventional ideas. The team can then agree to approach clinicians who hadn’t been involved with medical education before and who in our experience are often too willing to be part of the introduction of the new curriculum into their specialty.

### Tip 2: Encourage transparent ownership and accountability and use GANTT charts as a project management tool

A comprehensive discussion of project management is clearly beyond the scope of this article but a brief reflection is worthwhile. Outside academia, issues around communication and accountability are often regarded as key reasons why large projects fail. Overly complex or unclear management structures are also often implicated. For us as an institution it was also important to keep project management simple since nobody in the educational leadership team had any formal qualification or training in managing large scale projects. We have had great experience with GANTT charts to manage large scale change: Named after Henry Gantt, this is a chart that contains elements of a large project, together with agreed action points and ownership. In its initial incarnation ( Clark, Polakov, & Trabold, 1922), the GANTT chart was actually a production and shop-keeping tool ( Wilson, 2003). Its beauty is that even large projects with a multitude of work-streams and stakeholders can be broken down into individual action points in a GANTT chart. We now use Excel™ GANTT charts as a tool to manage most projects, such as the work process prior to the start of every new academic year. We keep these in a shared location and make them the focal point and update during work group meetings. GANTT charts can also cause friction in teams, usually when ownership or accountability are unclear or when there is overlapping ownership or competition for one aspect of the process. Again the importance of the GANTT chart lies in agreeing accountability and timelines.

### Tip 3: Define measurable outcomes, set realistic targets and agree time lines

In addition to the issues discussed in Tip 2, problems around poorly defined outcomes, unrealistic targets and lack of clear time lines are also common causes of why large scale projects fail. Others have emphasised that it is very difficult to implement any plan without knowing in advance what success looks like ( Paris, 2000). Measurable outcomes are extremely important in any plan, reflecting the purpose and the mission as a whole, allowing the team and individual team members to remain on track. In our experience it is very important to define early on what success looks like for each part of the plan and for every member of the team, and to agree transparent outcome measures.

Targets and time lines are just as important. In our experience leadership teams, when first faced with the task of driving large scale change, will often prescribe wildly unrealistic targets and time lines. Again GANTT charts are our tool of choice, keeping the team on task and giving workable portions of the plan to work on.

Regular meetings focus around the GANTT chart and not only action points, but also time lines can be agreed or changed. It is crucial that all team members feel able to speak up if things don’t go to plan and a previously agreed time line is no longer feasible. In this way the GANTT charts also serve as an early warning system to the leadership team who can then decide on whether to add more resources or modify targets.

### Tip 4: Think about what motivates tutors, identify the right people, and do not exclude clinical areas solely because they are busy

Our previous work around teaching awards ( Newton, Lewis, Pugh, Paladugu, & Woywodt, 2016) led us to understand that our clinical educators don’t seem to be driven by financial rewards or accolade. Instead, wanting to teach as a personality trait, coupled with perhaps idealism and enthusiasm for the tutor’s own specialty seemed to be key. Our awards system also plays to the competitive personality of our tutors and showcases those individuals who go the extra mile ( Newton et al., 2016). Our entire awards system costs as little as £1000 per year and we propose that even a Medical School in a developing country could set up a similar scheme at very little cost.

It was extremely important for the Working Group to identify the right clinicians to take on the challenge of change at the outset. With the short timescale, people with a ‘can do’ attitude were required and the Working Group identified such “Change Agents” ( Paton & McCalman, 2008) within each specialty, who were best placed to move the changes forward.

In our recruitment drive, one constant question was how to recruit additional clinical areas, i.e. those we had not utilised for student placements before. Managers and clinicians often label areas as “too busy for teaching” and medical admissions units (MAUs) and intensive care units (ICUs) are good examples. We have learned, through recruiting our own MAU ( Nazir et al., 2014) and ICU ( Khan et al., 2016) that with careful planning and supervision even extremely busy clinical areas can be very rewarding educationally ( Nazir et al., 2014) ( Khan et al., 2016).

### Tip 5: Invest in faculty development, work on team spirit and align educational aims with tutors’ appraisals

Identifying and recruiting enthusiastic tutors is a great starting point but by itself insufficient to deliver sustainable change. Enthusiastic tutors need to be supported and develop a sense of belonging and group identity. We hold regular tutors days during which we provide a mix of presentations and discussions but also the opportunity to reflect and float new ideas. This helps enormously in a large and growing teaching hospital where some of our tutors may not have met before.

These meetings foster group identity and bring people with similar degrees of enthusiasm for education together. Importantly, such events don’t require enormous resources and do not incur cost as such. We are also extremely keen that all of our long term tutors achieve Honorary Lecturer or Senior Lecturer status. We found this another great and cost-free way to build team spirit and a sense of community. Finally, winners of our teaching awards take home a statuette as a visible token of their achievement and thus act as ambassadors of our educational team ( Newton et al., 2016).

Finally, we have learned to utilise educational appraisals as an important part of our strategy: These annual 1:1 meetings between placement supervisors and a member of the leadership team are a fantastic opportunity to review achievements and build a loyal, dedicated and enthusiastic faculty. At no additional cost and with only a small impact on the workload of the leadership team, these appraisals will also help individuals to have their educational commitments recognised in their job plan. This will help to create a faculty that will be resilient even during times of austerity and high workload.

### Tip 6: Recognise individuality, provide outlets for enthusiasm and maximise non-monetary ways of supporting your tutors

Within any medical organisation there are many different strands of specialties and teams, all working in slightly different ways and often with their own very distinct team culture. We aim to recognise individuality and, where possible, allow placement providers to find their own solutions to problems. In our organisation one example is our surgical teaching where we allow the surgical secretaries to organise students timetables: While not in line with our educational structure overall, this has always worked well and allows the timetables to be synchronised with the commitments of our surgical colleagues in clinic and in theatres.

We have learned from our teaching awards ( Newton et al., 2016) that enthusiasm lies at the heart of why our tutors teach and we constantly try and provide outlets for educational enthusiasm and creativity. Educational projects and publications are useful and particularly projects that allow enthusiastic clinicians to showcase their specialty: We have launched a series of undergraduate workshops where enthusiastic clinicians showcase their specialty to interested students and thereby help students with career choice.

In addition to the suggestions in Tip 5, we are constantly trying to maximise non-monetary ways to support our tutors. In our experience, meeting all placement providers face-to-face was the most advantageous. These regular meetings allow for two-way discussions to take place, ensuring full understanding of the requirements. These meetings also feed into the annual appraisals and also give the leadership insight into placement providers’ clinical pressures and challenges outside medical education.

### Tip 7: Understand all funding streams related to undergraduate medical education in your institution

In the UK, a Service Increment for Teaching (SIFT) is paid to teaching Hospitals who provide Undergraduate placements. The payments form part of a teaching hospitals contract known as The Learning and Development Agreement (LDA). This LDA is put in place to ensure SIFT is utilised to pay for all areas of medical education. We cannot disclose exact amounts of funding but clearly a substantial amount of educational funding comes into our institution every year. Roughly 80% of SIFT funds posts, such as placement providers in the clinical workplace, placement support staff and coordinators and administrators. 20% funds the educational infrastructure and facilities including library services, office equipment, student accommodation and transportation. Importantly, our institution faced a 1.9% cut in SIFT funding in FY 2015/16 and will have to manage a further 2% cut in FY 2016/17. Much of our SIFT funded budget had grown organically over a long period of time and additional posts had been funded with the intention of fostering education. However many of these posts had attracted continued funding without regular scrutiny or performance review, as long as overall feedback for education was good. The envisaged cuts in our SIFT funding prompted us to start thinking about funding streams in much more detail. We began by looking into the entire SIFT income and came up with a very detailed list of expenditure which included salaries, but also other expenses, from subscriptions to equipment and facilities. One of the most surprising findings of our review was that some educational funds were used for posts that contributed very little to education, such as clinical staff or consultant secretaries.

### Tip 8: Align funding streams with educational delivery and aim for an agreed educational tariff

By its nature, budgeting is the responsibility of people ( Hannagan & Bennett, 2008) and once we had understood educational funding in much more detail, we set out to align this funding with delivery. Posts that contributed nothing to education, such as consultant secretaries in clinical departments, were withdrawn and the resulting funds re-invested in educational posts. This was a delicate procedure but led to the surprising paradox that we were able to increase the number of placement support staff despite ongoing annual cuts to our budget. We also worked with the hospital management and the Medical Director to agree an educational tariff whereby every possible role in undergraduate or postgraduate education accounted for a defined amount of time in clinicians’ supporting professional activity (SPA) time within their job plans. This tariff aligns with educational requirements and also ensures equity and fairness whereby only current contribution to teaching is remunerated, but not historical achievements or roles that no longer exist. Importantly, all job plans and their educational SPA components are scrutinised annually and require evidence of actual delivery.

### Tip 9: Ensure that educational funding, tariff and delivery are clear and transparent to all involved

Once we had aligned educational funding to delivery we were keen to ensure that this process was transparent to all stakeholders, i.e. clinicians (including those who did not participate in education), clinical directors and managers. We were especially keen to publicise the educational tariff agreed with hospital management (Tip 8). Next, we were keen to develop our knowledge of actual teaching delivery, beyond a collation of data on all education lead roles and job plans. To this end we recorded the actual delivery of all educational sessions within each specialty and defined a standard sessional fee. Finally, funding and expected educational delivery was agreed with each hospital department. Formal sign-off with senior managers as well as clinical and educational leads ensured delivery against expectations and funding. From then on, actual delivery was monitored and compared to agreements. This process also allows for identification of shortfalls in capacity, quality issues or anticipated national developments.

### Tip 10: Use change to identify future leaders

Successful organisations have been described as incubators of leadership ( Kotter, 1996) and change can be seen as a great facilitator and driver of this process. It was exciting for us to see placement supervisors and leads becoming empowered, as their teams took on the challenge of the revised curriculum. Allowing each lead to work with their own teams, using their own style of leadership to move the changes forward at their own pace allowed for successful change and for future leaders to emerge. We propose that change is actually a much better environment to identify future leaders than a steady-state environment in the sense that suitable individuals can show their creativity, organisational skills but also resilience and flexibility during times of great change. To foster this process and to encourage successful placement supervisors to progress their careers we have recently established additional educational area lead roles. These roles only involve half a session per week and therefore involved very little cost. However the new posts allowed enthusiastic individuals within the organisation to work on interesting projects, practice leadership skills, and gain visibility as an educator and as educational leaders of the future.

### Tip 11: Reflect on team culture and foster collaboration and reflection

Differences in team culture help to explain why some teams perform differently from others ( Mullins 2013). However team culture can also be the barrier to change if teams are overly protective and reluctant to share knowledge or expertise with others. Some teams perceive change as a threat for fear that team members, ways of working, or processes are at risk. It is important to convey that change is inevitable and that it is preferable to be robust, pace-setters, leaders of change, rather than passive bystanders. With this positive attitude comes the open exchange of new ideas, exciting innovations and developments. It is important for educational institutions to consciously reflect on team culture and to factor in time for reflection. To this end we hold regular team away and lessons learned events and try to find out what was done well, what could have been done differently and what can be improved upon. For these events we take our teams away from their usual workplace to a room in a different part of the organisation. Discussing things that went well sends a very powerful message to see the connection between the change and the performance improvement ( Kotter, 1996). At very little additional cost, these regular events have been very important to foster team culture and to allow the wheels of change to turn a little easier next time.

### Tip 12: Encourage a culture of continuous evaluation and improvement and reward both team and individual achievements

In their landmark document *Tomorrows Doctors* the UK’s General Medical Council requested that “The medical school will have [..] systems to monitor the quality of teaching and facilities on placement”. ( GMC, 2009). In our institution both quantitative and qualitative data are sought regularly from the students at the end of each teaching block. All feedback is shared with placement supervisors at end of placement review meetings. Others have emphasised that it is imperative that individuals within organisations see the fruits of their labour ( Sutherland & Canwell, 2004). Placement supervisors are no exception to this and we take these face to face meetings very seriously: They include agreed minutes, praise for individuals and teams but also ideas on how the placement can be improved further. The requirement for continued improvement has been characterised as a necessary part of effective teamwork ( Mullins & Gill, 2013) and continuous striving to improve is often seen as key attribute of successful organisations ( Collins & Porras, 1994). Along the same line we have recently reported our favourable experience with turning already existing QA data into a system of teaching awards ( Newton et al., 2016). At very little additional cost, this development helped us enormously to foster a culture of continued improvement and reward individual and team achievement.

## Conclusion

Austerity is defined as difficult economic conditions created by government measures to reduce public expenditure. The effects of continued austerity on public health and healthcare in general have been described in great detail elsewhere ( Karanikolos et al., 2013) ( Aiken et al., 2014; Reeves, McKee, & Stuckler, 2015). Increasing demand from an ageing population and no growth in real-terms funding in 2018/19 and only 0.4 per cent in 2019/20 ( Maguire, Dunn, & McKenna, 2016) have led to the UK’s National Health Service facing a target of £22 bn in efficiency savings to address. Very little is known about the impact of economic austerity on medical education.

Firstly, ever-increasing clinical workload and time pressures may lead to declining staff morale with cynicism and burnout and it is also easy to see how, in a system under pressure, educational activities may have less priority than direct patient care. Secondly, such a situation may potentially lead to a “brain drain” with highly qualified academic clinicians moving to countries with more advantageous economic climate. Such an effect is well described for countries such as Greece ( Trachana, 2013) ( Ifanti, Argyriou, Kalofonou, & Kalofonos, 2014) and Croatia ( Bojanic, Bojanic, & Likic, 2015). There is some evidence of a similar effect in the UK: In 2013, out of the 12231 medical practitioners who returned their license to the General Medical Council, 6886 did so to work abroad ( GMC, 2014). It is not inconceivable that such a brain drain includes highly motivated individuals who, had they stayed, would have contributed substantially to undergraduate medical education. Thirdly, for an institution such as ours i.e. a large but non-University teaching hospital there is also a strategic risk: Medical schools can, at least to some degree, choose between surrounding hospitals when it comes to providing clinical placements. In doing so they place more and more demands on placement providers, flanked by more and more detailed quality assurance and in-depth student feedback. In the worst case scenario our institution would risk losing a substantial stream of income if deteriorating student feedback led to withdrawal of students by the Medical School.

Faced with these clear risks, our little report is a toolkit for educational teams who must deliver substantial change despite financial austerity. In our experience maintaining good and supportive professional relationships with a teaching faculty under considerable pressure was crucial to our success. We were able to drive substantial change, recruit numerous clinicians who were previously not involved in undergraduate education, and improve the quality of education overall. We propose that, while economic austerity remains a challenge to educational teams, it also poses opportunities: Given the choice between additional clinical workload and educational commitments enthusiastic individuals are easily tempted towards the latter. It is also worthwhile to remember that, within limits, workload does not automatically impair teaching ( Robinson, 2015). Finally, clinicians may have concerns over their long term career and developing an educational profile within the organisation can be an attractive proposal.

Our report has limitations as well: What looks like severe austerity to an educational team in the UK may be regarded as generous funding elsewhere. Furthermore, some of our tips rely on particulars of the UK’s medical system such as formal agreed job plans, which may not exist elsewhere. We are very conscious of the limitations of our article and we tried to formulate tips that would work elsewhere and regardless of the level of initial funding: An awards scheme, for example, will cost very little yet in our experience achieve a lot if well designed, regardless of the order of magnitude of financial austerity. Similarly, a sense of community and team spirit among educators can be generated regardless of the medical system or level of funding overall.

In summary, our experience has been very rewarding and positive, despite our own initial concerns and widespread fears within the team. In essence, we turned a period of financial austerity into an opportunity to improve the teaching for our students. This experience has also made us a lot more confident and resilient. One key factor was the notion that tutors are motivated by non-monetary factors. We propose that educators need to focus on and nurture these non-monetary factors when they face austerity. Equally important was the desire for the leadership team to understand the flows of educational money, interact with managers, and align funding with educational delivery. We have also learned a lot about change within an organisation and how to communicate, drive, and consolidate it. We would encourage others faced with similar challenges to consciously reflect on the process, try out our suggestions, and turn financial austerity into an opportunity.

## Take Home Messages

• With increasing financial demands on educational teams, be mindful of the non-monetary opportunities that can arise from substantial change and remember to reward the enthusiastic teacher.

• Always keep the lines of communication open between teams, allowing ideas to flow and encouraging empowerment.

• Keep your change management on track by utilising the right tools for the job and maintaining realistic targets.

• A busy workload doesn’t always impair teaching. Good planning and clear supervision can make even the busiest of clinical areas rewarding places to learn.

• Don’t be afraid to consciously reflect and to learn lessons that will allow future improvement and stronger teams.

## Notes On Contributors

Kay Cartwright is Medical Education Manager at Lancashire Teaching Hospitals NHS Foundation Trust.

Paladugu Madhavi is a Consultant Paediatrician and Hospital Dean at Lancashire Teaching Hospitals NHS Foundation Trust.

Keiarash Kazemi-Jovestani is Associate Undergraduate Dean for Year 4 at Lancashire Teaching Hospitals NHS Foundation Trust.

Susan Maxwell is Head of Business and Operations Lancashire Teaching Hospitals NHS Foundation Trust.

Maggie Wilson is Head of Educational Performance and Quality Assurance at Lancashire Teaching Hospitals NHS Foundation Trust.

Alexander Woywodt is a Consultant Nephrologist and Associate Undergraduate Dean at Lancashire Teaching Hospitals NHS Foundation Trust.
