# Supplementary material for: Twelve Tips to implement Curriculum Changes in times of Economic Austerity
Source: MedEdPublish (2016). 2017 Mar 9;6:47. [Version 1] doi: 10.15694/mep.2017.000047 (PMC10885242; doi:10.15694/mep.2017.000047)
Supplement: Peer review history

# Reviewer report: Reviewer response for version 1

**DOI:** 10.21956/mep.19452.r28655

**Published:** 2017-03-10

**Authors:** John Cookson

**Affiliations:** 1 University of Worcester

This review has been migrated. The reviewer awarded 4 stars out of 5 Agree with the others. In the UK system, a close relationship between the SIFT finding and actual timetabled teaching and job plans is the key to success

Reviewer Expertise:

NA

No decision status is available

# Reviewer report: Reviewer response for version 1

**DOI:** 10.21956/mep.19452.r28653

**Published:** 2017-03-10

**Authors:** James Fraser

**Affiliations:** 1 Griffith University

This review has been migrated. The reviewer awarded 4 stars out of 5 The author’s reflections on their recent experiences of curriculum change are consistent with much of the cross disciplinary literature in change management and would provide a framework for those tasked with similar challenges in medical curricula. A number of the tips importantly focus on communication, connection, culture and teaching staff motivation and skill development as being keys to successful change. In keeping with the focus of the paper the authors discuss factors that may be addressed by provision of opportunities that don’t directly impact limited finances an evaluation of staff acceptance and satisfaction of these would be an interesting extension of this work.It would also be interesting to understand which of the tips were applicable to receiving student input and any benefits or concerns that arose as a result.

Reviewer Expertise:

NA

No decision status is available

# Reviewer report: Reviewer response for version 1

**DOI:** 10.21956/mep.19452.r28652

**Published:** 2017-03-09

**Authors:** Michelle McLean

**Affiliations:** 1 Bond University

This review has been migrated. The reviewer awarded 4 stars out of 5 Cartwright and colleagues' Twelve Tips article reminds us of a number of important principles around change management, i.e. good leadership, vision, transparency, inclusiveness, the right culture. None of this is new, but having read about some individuals’ ‘difficult circumstances’ over the past year (e.g. a toxic institutional culture), Cartwright et al’s advice is a timely reminder as most of us are experiencing budget cuts and increased student and patient numbers. An important point that the authors make is the need to identify the right person for the job (Tip 4). If everyone plays to his/her strengths, the team must move forward and be productive. This point is taken further in Tip 6, where the authors cite their own work about monetary reward is not being the be-all and end-all. The right person will be enthusiastic and motivated and will deliver and possibly change attitudes. Once again, thanks for reminding us.

Reviewer Expertise:

NA

No decision status is available

# Reviewer report: Reviewer response for version 1

**DOI:** 10.21956/mep.19452.r28654

**Published:** 2017-03-09

**Authors:** Trevor Gibbs

**Affiliations:** 1 AMEE

This review has been migrated. The reviewer awarded 4 stars out of 5 Its important to think about what other factors come into play when structuring or re-structuring a curriculum; a very common factor relates to financial resources. This is a very useful paper that covers the experience of a UK based team re-designing a particular part of an undergraduate medical curriculum and as such provides some very good tips to those new to the activity. Hence I would recommend this paper to those who are considering such change.Much is made about the opportunities that arise that may not be directly related to finances and monetary rewards. I would have liked these to be explained a little more because I am personally not sure that these are sustainable- I would however bow to the authors recent experience in this area. Perhaps an extended evaluation is required here, with special reference to the take home message that " A busy workload doesn't always impair teaching. Good planning and clear supervision can make even the busiest of clinical areas rewarding places to learn."

Reviewer Expertise:

NA

No decision status is available
